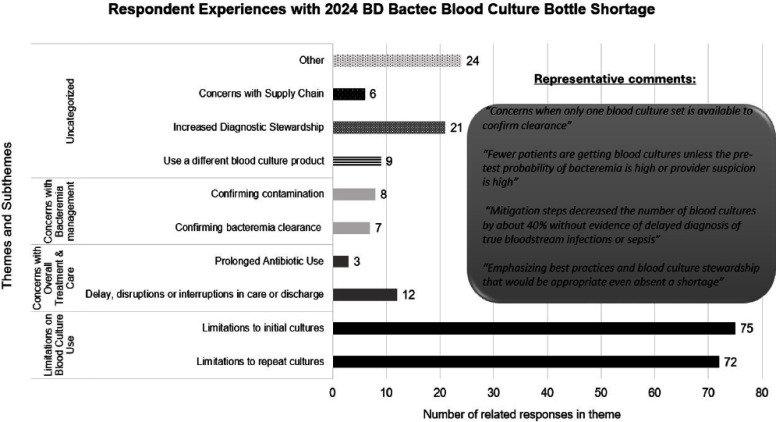# Turning Crisis into Opportunity: Blood Culture Stewardship and the National Impact of a Blood Culture Bottle Shortage on Clinical Care

**DOI:** 10.1017/ash.2025.318

**Published:** 2025-09-24

**Authors:** Evangeline Green, Jonathan Ryder, Harlan Sayles, Susan E. Beekmann, Philip Polgreen, Jasmine Marcelin

**Affiliations:** 1 University of Nebraska Medical Center; 2 University of Iowa; 3 University of Iowa Hospitals and Clinics

## Abstract

**Background:** In 2024, US hospitals were affected by the Becton Dickinson (BD) BACTEC blood culture bottle shortage with little time to respond and conserve supply. The extent of the impact of this shortage on clinical practice has not been explored. **Methods:** We developed a 7-question online poll with the Emerging Infections Network (EIN) exploring the extent to which facilities were impacted by the shortage, geographic distribution and facility type of institutions affected, actions taken to mitigate the shortage, and the impact on clinical management of fever and Staphylococcus aureus bacteremia. The link was sent to >3100 EIN listserv members 3 times during September 2024. Descriptive and thematic analyses were performed on quantitative and qualitative responses. **Results:** Of 202 respondents from 39 states, 129(64%) responded their hospital had limited blood cultures available, 8(4%) were unsure how their hospitals were affected, and 65(32%) indicated their hospitals were not affected (Fig1). The most affected hospital facility types with >10 respondents were Community (27/39, 69%), University (48/72, 67%), Children’s (7/11, 67%), Non-university teaching (33/52, 65%), and the VA/DOD was least affected facility type (3/11, 27%). Respondents not affected by the shortage most commonly used alternate blood culture media. Top mitigation strategies included publishing algorithms for best practice use (103/202, 51%), restricting follow-up blood cultures (88/202, 44%), using single blood culture sets (86/202, 43%), and implementing EMR-based alerts on blood culture orders (71/202, 36%). Important clinical themes identified by affected respondents included limitations on blood culture use (147 responses), concerns with overall treatment and care including delays and disruptions in discharges or prolonged antibiotic use (15 responses), concerns with bacteremia management (15 responses), and increased diagnostic stewardship opportunities (21 responses) (Fig2). The most prevalent theme in S. aureus bacteremia management was limitations in repeat blood cultures (61/163, 37%) with concerns about confirming bacteremia clearance, while the most common theme in inpatient/ER management of fever was limitations in initial blood cultures (64/159, 40%), with common comments about reducing inappropriate blood cultures. 61/202 respondents commented in the open-ended question with the most common theme highlighting increased diagnostic stewardship as a positive outcome of the shortage (19/61, 31%). **Conclusion:** The BD BACTEC blood culture bottle shortage caused widespread clinical impact. The themes identified highlight the challenges placed on healthcare systems during times of shortage as well as the effects on patient care. Mitigation strategies implemented during the shortage may create future opportunities for diagnostic stewardship.

**Figure:**
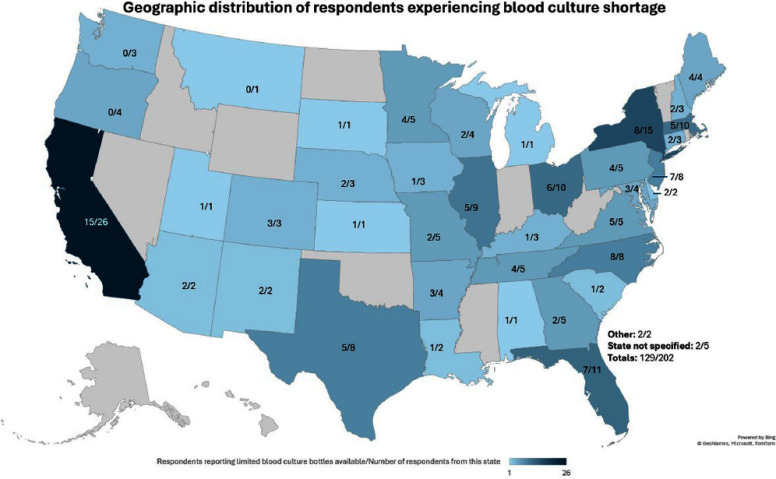

**Figure:**